# Genome-wide methylation sequencing identifies progression-related epigenetic drivers in myelodysplastic syndromes

**DOI:** 10.1038/s41419-020-03213-2

**Published:** 2020-11-20

**Authors:** Jing-dong Zhou, Ting-juan Zhang, Zi-jun Xu, Zhao-qun Deng, Yu Gu, Ji-chun Ma, Xiang-mei Wen, Jia-yan Leng, Jiang Lin, Su-ning Chen, Jun Qian

**Affiliations:** 1 Department of Hematology, Affiliated People’s Hospital of Jiangsu University, Zhenjiang, Jiangsu People’s Republic of China; 2 Zhenjiang Clinical Research Center of Hematology, Zhenjiang, Jiangsu People’s Republic of China; 3 The Key Lab of Precision Diagnosis and Treatment in Hematologic Malignancies of Zhenjiang City, Zhenjiang, Jiangsu People’s Republic of China; 4 Laboratory Center, Affiliated People’s Hospital of Jiangsu University, Zhenjiang, Jiangsu People’s Republic of China; 5 Jiangsu Institute of Hematology, National Clinical Research Center for Hematologic Diseases, NHC Key Laboratory of Thrombosis and Hemostasis, The First Affiliated Hospital of Soochow University, Suzhou, People’s Republic of China; 6 Collaborative Innovation Center of Hematology, Soochow University, Suzhou, People’s Republic of China

**Keywords:** DNA methylation, Predictive markers, Prognostic markers, Myelodysplastic syndrome

## Abstract

The potential mechanism of myelodysplastic syndromes (MDS) progressing to acute myeloid leukemia (AML) remains poorly elucidated. It has been proved that epigenetic alterations play crucial roles in the pathogenesis of cancer progression including MDS. However, fewer studies explored the whole-genome methylation alterations during MDS progression. Reduced representation bisulfite sequencing was conducted in four paired MDS/secondary AML (MDS/sAML) patients and intended to explore the underlying methylation-associated epigenetic drivers in MDS progression. In four paired MDS/sAML patients, cases at sAML stage exhibited significantly increased methylation level as compared with the matched MDS stage. A total of 1090 differentially methylated fragments (DMFs) (441 hypermethylated and 649 hypomethylated) were identified involving in MDS pathogenesis, whereas 103 DMFs (96 hypermethylated and 7 hypomethylated) were involved in MDS progression. Targeted bisulfite sequencing further identified that aberrant *GFRA1*, *IRX1*, *NPY*, and *ZNF300* methylation were frequent events in an additional group of de novo MDS and AML patients, of which only *ZNF300* methylation was associated with *ZNF300* expression. Subsequently, *ZNF300* hypermethylation in larger cohorts of de novo MDS and AML patients was confirmed by real-time quantitative methylation-specific PCR. It was illustrated that *ZNF300* methylation could act as a potential biomarker for the diagnosis and prognosis in MDS and AML patients. Functional experiments demonstrated the anti-proliferative and pro-apoptotic role of *ZNF300* overexpression in MDS-derived AML cell-line SKM-1. Collectively, genome-wide DNA hypermethylation were frequent events during MDS progression. Among these changes, *ZNF300* methylation, a regulator of *ZNF300* expression, acted as an epigenetic driver in MDS progression. These findings provided a theoretical basis for the usage of demethylation drugs in MDS patients against disease progression.

## Introduction

Myelodysplastic syndromes (MDS) comprise a diverse group of clonal hematopoietic disorders characterized by peripheral blood cytopenias and ineffective production of blood cells^1^. Although the intensive treatments are carried out appropriately, ~30% of MDS patients will progress to acute myeloid leukemia (AML) with poor prognosis^1,2^. However, the potential mechanism of MDS progression remains poorly investigated. Accumulated pre-existing genetic/epigenetic abnormalities and newly emerging events play crucial roles in the progression of MDS. Thus, identification of specific molecular events could make better understanding of MDS pathogenesis and may guide clinical treatment against disease progression.

With a rapid advance in sequencing methodologies, whole-genome or whole-exome sequencing have been successively utilized to identify huge genetic changes in MDS^3–7^. Gene mutations in functional gene categories, including epigenetic modifiers (*TET2* and *IDH1/2*), RNA splicing machinery (*ASXL1*), transcriptional factors (*RUNX1*), and signal transduction factors (*ROBO1/2*), have been considered to be partially responsible for progression in MDS^3–7^. These genetic drivers, however, could not generalize all the cases during MDS progression. The frequently mutant genes *TET2* and *IDH1/2* in MDS-derived secondary AML (sAML) are also methylation-related genes suggesting that aberrant epigenetic programming may play a crucial role in MDS progression^8^. Previously, we have already identified that single genes, including *GPX3*, *ID4*, and *SOX30* methylation were associated with disease progression in MDS^9–11^. To gain new insights into the epigenetic mechanism underlying disease evolution in MDS, reduced representation bisulfite sequencing (RRBS) was conducted in bone marrow (BM) samples from four paired MDS/sAML patients and intended to discover methylation-associated epigenetic drivers in MDS progression.

In the current study, we investigated genome-wide methylation of four paired MDS/sAML patients by RRBS, and confirmed increased genomic hypermethylation as a frequent phenomenon during MDS progression. Secondly, several genes methylation patterns were screened and verified in MDS and AML patients using targeted bisulfite sequencing. Simultaneously, real-time quantitative methylation-specific PCR (RQ-MSP) was implemented to detect the identified gene methylation pattern in a larger group of clinical samples. Finally, the potential role and biological network of the targeted gene were further determined in MDS-derived AML cell-line SKM-1^12,13^. The current work demonstrated a novel contribution to the epigenetic alterations profile of MDS during disease progression and could be potentially helpful to guide treatment decisions for MDS against disease progression.

## Materials and methods

### Patients and samples

Firstly, the BM samples from four paired MDS/sAML patients and four healthy donors were obtained in the Affiliated People’s Hospital of Jiangsu University and the First Affiliated Hospital of Soochow University. No significant differences were observed in age of controls (from 47 to 58 years old) and paired MDS/sAML patients (from 36 to 74 years old) (*P* = 0.828). All four paired MDS/sAML patients did not receive any demethylation treatment such as azacitidine and decitabine before. The detail information of four paired MDS/sAML patients was presented in Table S1. Secondly, another cohort of samples was enrolled for targeted bisulfite sequencing consisting of 35 de novo MDS, 111 de novo AML patients and 25 healthy donors treated at the Affiliated People’s Hospital of Jiangsu University. Furthermore, a total of 70 de novo MDS and 170 de novo AML patients together with 46 healthy donors treated at the Affiliated People’s Hospital of Jiangsu University were also included in validation assays. The diagnosis and classification of MDS and AML patients were established mainly according to the revised World Health Organization (WHO) criteria^14^. Common gene mutations of MDS and AML patients were detected as our previous report^10^. Treatment regimens for MDS and AML patients were reported previously as well^10^. BM was collected from all of the subjects after providing written informed consents. BM mononuclear cells (BMMNCs) were separated using Lymphocyte Separation Medium (Solarbio Science & Technology Co., Ltd., Beijing, China) based on density-gradient centrifugation, and RNA and DNA extraction were carried out subsequently^15^. This study was approved by the Ethics Committee of Affiliated People’s Hospital of Jiangsu University.

### RRBS

RRBS was performed in Genesky Biotechnologies Inc. (Shanghai, China). Genomic DNA from BMMNCs was extracted and digested with MspI enzyme. After end-repair and A-tailing, methylated adaptors were ligated to MspI fragments. Adaptorligated fragments were size-selected (150–350 bp), bisulfite converted using EZ DNA Methylation kit and PCR amplified (12–15 cycles). After purification by TIANquick Midi Purification Kit, libraries were quantified and the quality was assessed. Single-ended RRBS libraries with 2 × 150 bp read length were sequenced on an Illumina HiSeq2500. The sequenced reads were mapped against the complete human reference genome GRCh37/h19 using the Bismark alignment tool.

### Targeted bisulfite sequencing

DNA methylation of target genes was evaluated by Targeted Bisulfite Sequencing (MethylTarget) performed in Genesky Biotechnologies Inc. (Shanghai, China). The primers used for selected genes were listed in Table S2. The MethylTarget assay was described clearly as reported^16,17^.

### Reverse transcription and RQ-PCR

Reverse transcription was carried out using random primers as reported^15,16^. Determination of selected gene expression was detected by real-time quantitative PCR (RQ-PCR) using AceQ qPCR SYBR Green Master Mix (Vazyme Biotech Co., Piscataway, NJ). *ABL1* examined by 2×SYBR Green PCR Mix (Multisciences, Hangzhou, China) was applied to calculate the abundance of mRNA expression. The primers used for selected genes were given in Table S2. Relative mRNA expression was calculated by 2^−∆∆CT^ method.

### Bisulfite modification and RQ-MSP

Genomic DNA was bisulfite converted as our previous report^15,16^. The methylation level of selected genes was detected by RQ-MSP with primers listed in Table S2. *ALU* was utilized to calculate the abundance of gene methylation level. Relative gene methylation level was calculated using 2^−∆∆CT^ method.

### Cell lines and cell culture

The MDS-derived AML cell-line SKM-1 were cultured in RPMI 1640 medium containing 10% fetal calf serum (ExCell Bio, Shanghai, China) and grown at 37 °C in 5% CO_2_ humidified atmosphere^12,13^.

### Demethylation studies

SKM-1 cells at a density of 5 × 10^5^ cells/ml in 2 ml were treated with 5-aza-2′-deoxycytidine (5-aza-dC) with a final concentration of 0 μM, 1 μM, 2 μM, and 10 μM during 4 days.

### Cell transfection

Human full-length *ZNF300* CDS sequences were introduced into the BamHI/AgeI of GV569 (Ubi-MCS-3FLAG-CBh-gcGFP-IRES-puromycin) vector (GENECHEM, Shanghai, China), and were transfected with lentivirus (Lv).

### Western blot analysis

Western blot was carried out as described^10,18^. The antibodies were rabbit anti-ZNF300 (Sigma-Aldrich, St. Louis, MO) and mouse anti-GAPDH (BOSTER, Wuhan, China).

### Cell growth assays

The transfected cells were seeded in 96-well plates at a density of 5 × 10^3^ cells per well in triplicate. After culture for 0 h, 24 h, 48 h, 72 h, and 96 h, CCK-8 was added to each well and incubated for 2 h. The absorbance at 450 nm was measured using a microplate reader. The rate of cell growth was calculated as OD value.

### Cell apoptosis analysis

The transfected SKM-1 cells were cultured with serum-free medium for 48 h in 6-well plates (5 × 10^5^ cells/well). Annexin V-APC Kit was used to analyze the apoptosis rate according to the manufacturer’s protocols and then analyzed via flow cytometry.

### Cell cycle analysis

The transfected SKM-1 cells were harvested after 48 h incubation with a density of 5 × 10^5^ cells per well in 6-well plates in triplicate. After fixed in 70% ice-cold ethanol in PBS, the cells were washed twice and added with propidium iodide to analyze the cell cycle distribution according to the manufacturer’s protocols via flow cytometry.

### Statistical analysis

SPSS 22.0 and GraphPad Prism 5.0 were the main software package in statistical analysis. Independent T/Paired T/Mann–Whitney’s *U*-tests were applied for the comparison of continuous variables, whereas Pearson Chi-square/Fisher exact tests were used for the comparison of categorical variables. Spearman correlation test was performed to analysis the correlation between *ZNF300* expression and methylation. The receiver operating characteristic (ROC) curve and area under the ROC curve (AUC) were applied to assess the discriminative capacity of *ZNF300* methylation between AML patients and controls. The impact of *ZNF300* methylation on complete remission (CR) was tested by univariate and multivariate logistic regression models. The prognostic effects of *ZNF300* expression on leukemia-free survival (LFS) and overall survival (OS) were analyzed through Kaplan–Meier analysis (Log-rank test) and Cox regression analysis (univariate and multivariate proportional hazard regression). Two-tailed *P*-values < 0.05 in all analysis was considered as statistically significant differences.

## Results

### Genome-wide methylation analysis of paired MDS/sAML patients

To identify epigenetic alterations occurring in MDS progression, genome-wide methylation pattern was explored using RRBS in four newly diagnosed MDS patients and the matched patients at sAML stage. In addition, four healthy donors were also included as normal controls. The sequencing data were submitted to NCBI SRA databases with an accession number PRJNA670308. A mean of 2.2 × 10^7^ total pair reads per sample were produced from RRBS libraries and mapped to the reference human genome (UCSC hg19) using Bismark (http://www.bioinformatics.babraham.ac.uk/projects/bismark/). The mapping efficiency of sequenced reads was about 58% of each sample. A mean of 7.5 × 10^8^ cytosines were analyzed per sample and about 17% (1.2 × 10^8^) of these cytosines were located in CpG sites. RRBS data quality was shown in Table S3.

**Fig. 1:**
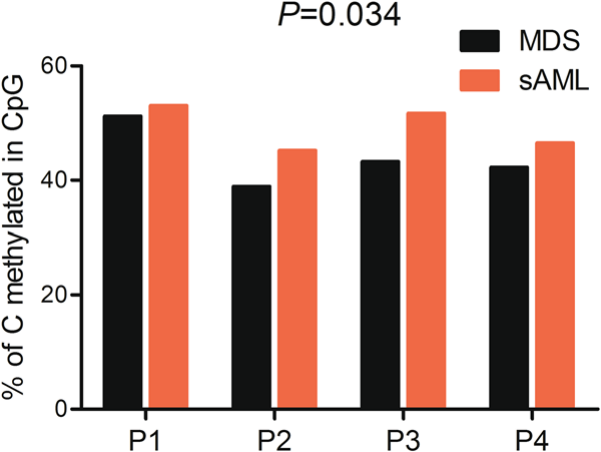
Whole-genome methylation profiling in four paired MDS/sAML patients. The percentage of methylated CpG sites in controls and MDS/sAML was demonstrated. *P*-values were calculated using the Paired *T*-test.

The mean global methylation in controls ranged from 45.4% to 51.0%, similar to the methylation patterns for MDS/sAML patients ranging from 36.8% to 53.1% (Table S3 and Fig. S1). However, in four paired MDS/sAML patients, cases at sAML stage exhibited significantly increased methylation level when compared with their matched MDS stage (Fig. 1). Hierarchical clustering of controls and MDS/sAML patients based on CpG site methylation separated MDS/sAML patients from controls (Fig. S1).

### Differential methylation landscapes in paired MDS/sAML patients

**Fig. 2:**
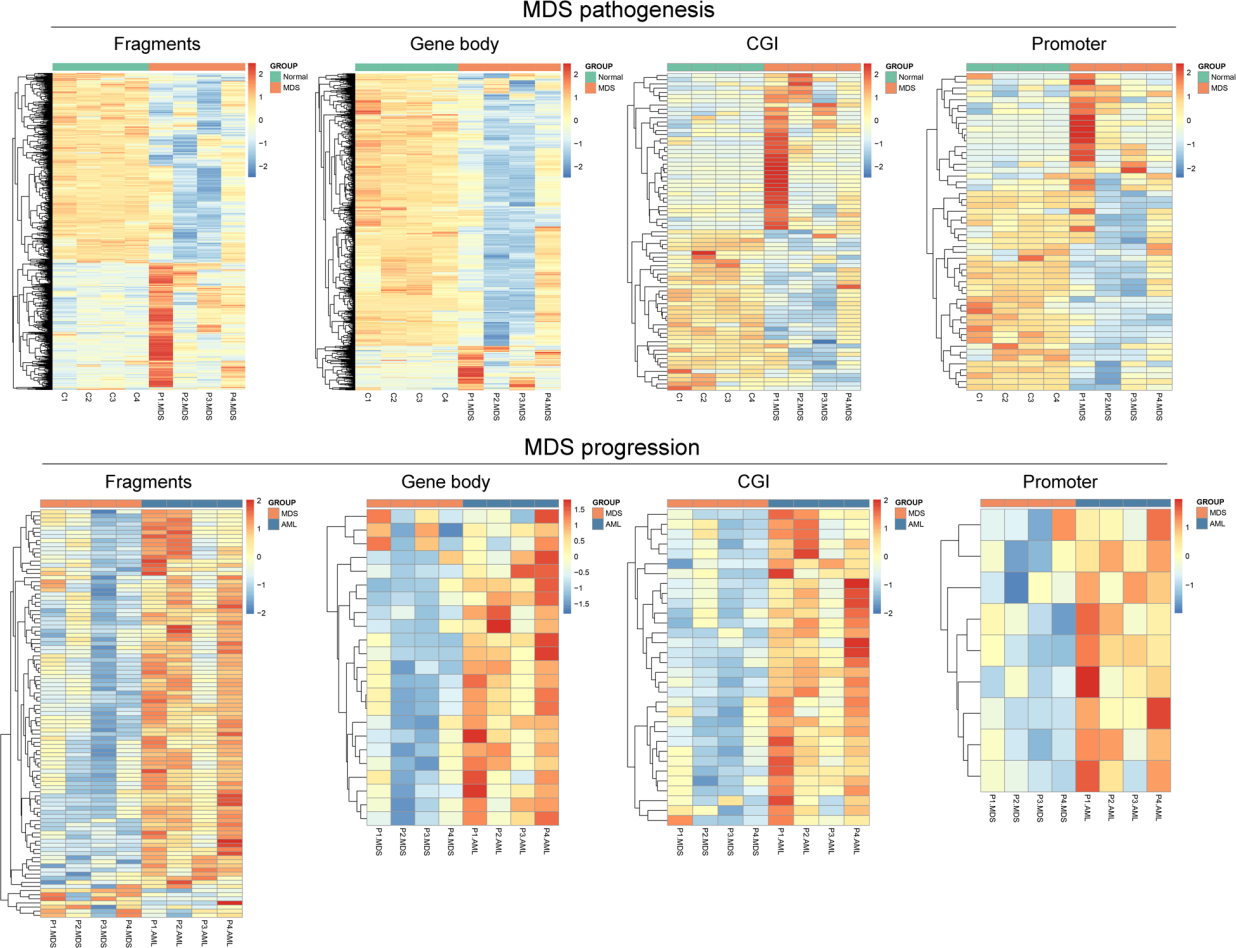
Heatmaps summarizing differentially methylated fragments/genes analyzed by the unit of *Mspl* fragments, CpG islands, gene body, and promoter. MDS pathogenesis indicated the difference compared between MDS and controls. MDS progression indicated the difference compared between sAML and MDS. The fragments/genes that passed statistical significance (paired/independent *T* test-*P* < 0.05, and also had >25% mean methylation difference) were considered as differentially methylated fragments/genes.

*Mspl* fragments (40–220 bp) rather than individual CpG sites or a tiled window approach were regarded as the basic analysis units as described previously^19–21^. The fragments that passed statistical significance (paired/independent *T* test-*P* < 0.05, and also had >25% mean methylation difference) were considered as differentially methylated fragments (DMFs). We identified 1090 DMFs (441 hypermethylated and 649 hypomethylated) between MDS and controls (MDS vs. controls), which may be seen as molecular events contributing to MDS pathogenesis. Moreover, a total of 103 DMFs (96 hypermethylated and 7 hypomethylated) were identified between sAML and MDS (sAML vs. MDS), seen as molecular events contributing to MDS progression (Fig. 2). A detailed description of these DMFs was given in Table S4 and Table S5.

Next, CGI, promoter (±2000 bp from transcription start site), and gene body were also used as the unit of analysis, respectively. For CGI, we identified 74 differentially methylated genes (DMGs) (37 hypermethylated and 37 hypomethylated) in MDS vs. controls, which may be involved in MDS pathogenesis, and 32 DMGs (32 hypermethylated) in sAML vs. MDS, involved in MDS progression (Fig. 2). Secondly, for promoter, we discovered 54 DMGs (22 hypermethylated and 32 hypomethylated) in MDS vs. controls and 9 DMGs (9 hypermethylated) in sAML vs. MDS (Fig. 2). Furthermore, for gene body, the numbers were 1023 DMGs (144 hypermethylated and 879 hypomethylated in MDS vs. controls) and 26 DMGs (23 hypermethylated and 3 hypomethylated in sAML vs. MDS) respectively (Fig. 2). More detailed descriptions of these DMGs were illustrated in Tables S4 and S5.

### Comparison of methylome of independent paired MDS/sAML patients

**Fig. 3:**
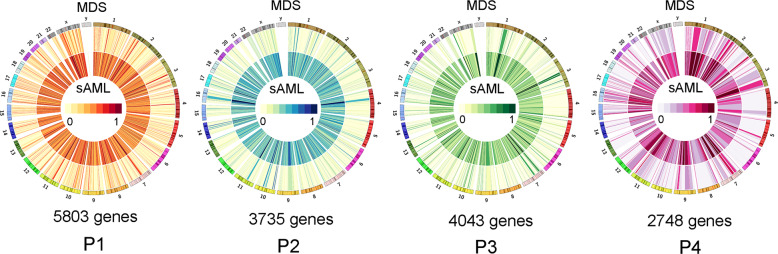
Differentially methylated genes in four paired MDS/sAML patients presented by circos plots. The circos plot represented methylation values in MDS/sAML patients. Outer ring indicated MDS sage, inner ring indicated sAML stage. The fragments/genes that passed statistical significance (paired/independent *T* test-*P* < 0.05, and also had >25% mean methylation difference) were considered as differentially methylated fragments/genes.

**Fig. 4:**
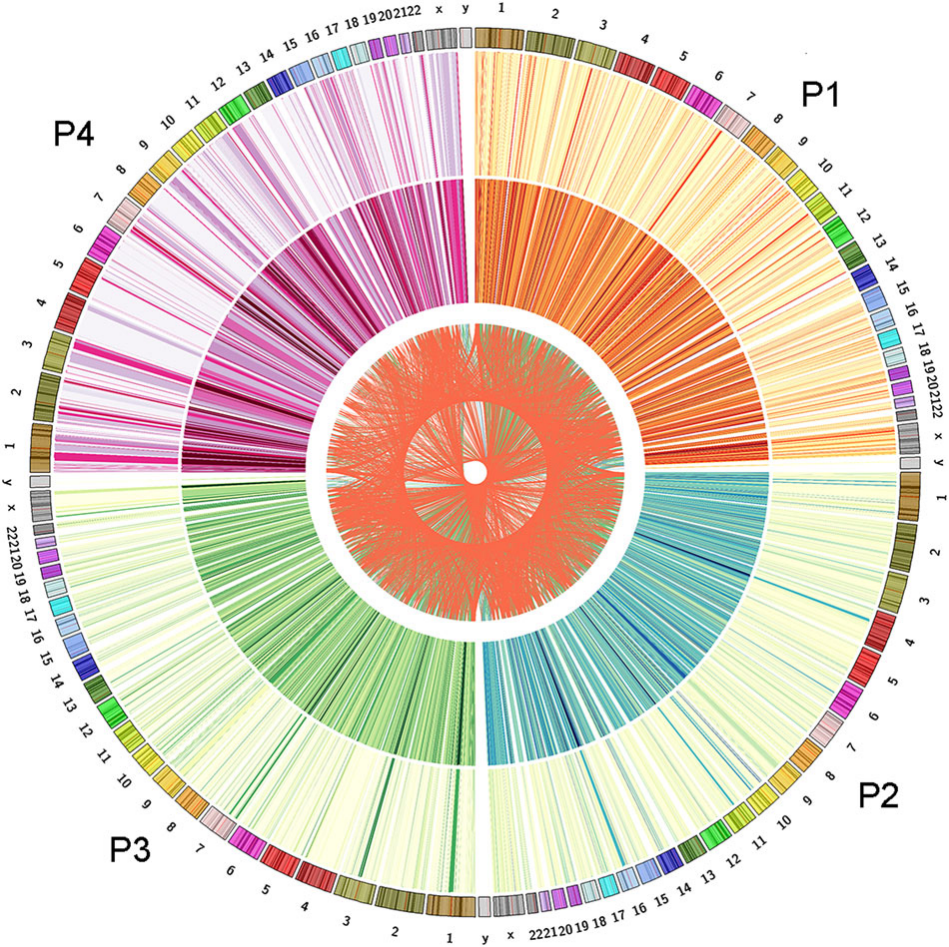
Overlap genes discovered to be differentially methylated in four paired MDS/sAML patients. The circos plot represented methylation values in MDS/sAML patients. Outer ring indicated MDS sage, inner ring indicated sAML stage. Inside lines indicated genes that differentially methylated in at least two of the patients. The fragments/genes that passed statistical significance (paired/independent *T* test-*P* < 0.05, and also had >25% mean methylation difference) were considered as differentially methylated fragments/genes.

Since each of the four paired MDS/sAML patients contained distinct epigenomes, we further performed differential methylation analysis on each pairs independently. Using the promoter as the unit of analysis, we identified 5803, 3735, 4043, and 2748 DMGs (fragments annotated as genes with >25% mean methylation difference) between MDS and sAML stage in four individual paired patients (Fig. 3). A total of 4558 genes were abnormally methylated in at least two of the samples, while 2130 genes in at least three of the samples and 634 genes in all four samples (Fig. 4).

### Identification and validation of DMFs by targeted bisulfite sequencing in MDS/AML patients

**Fig. 5:**
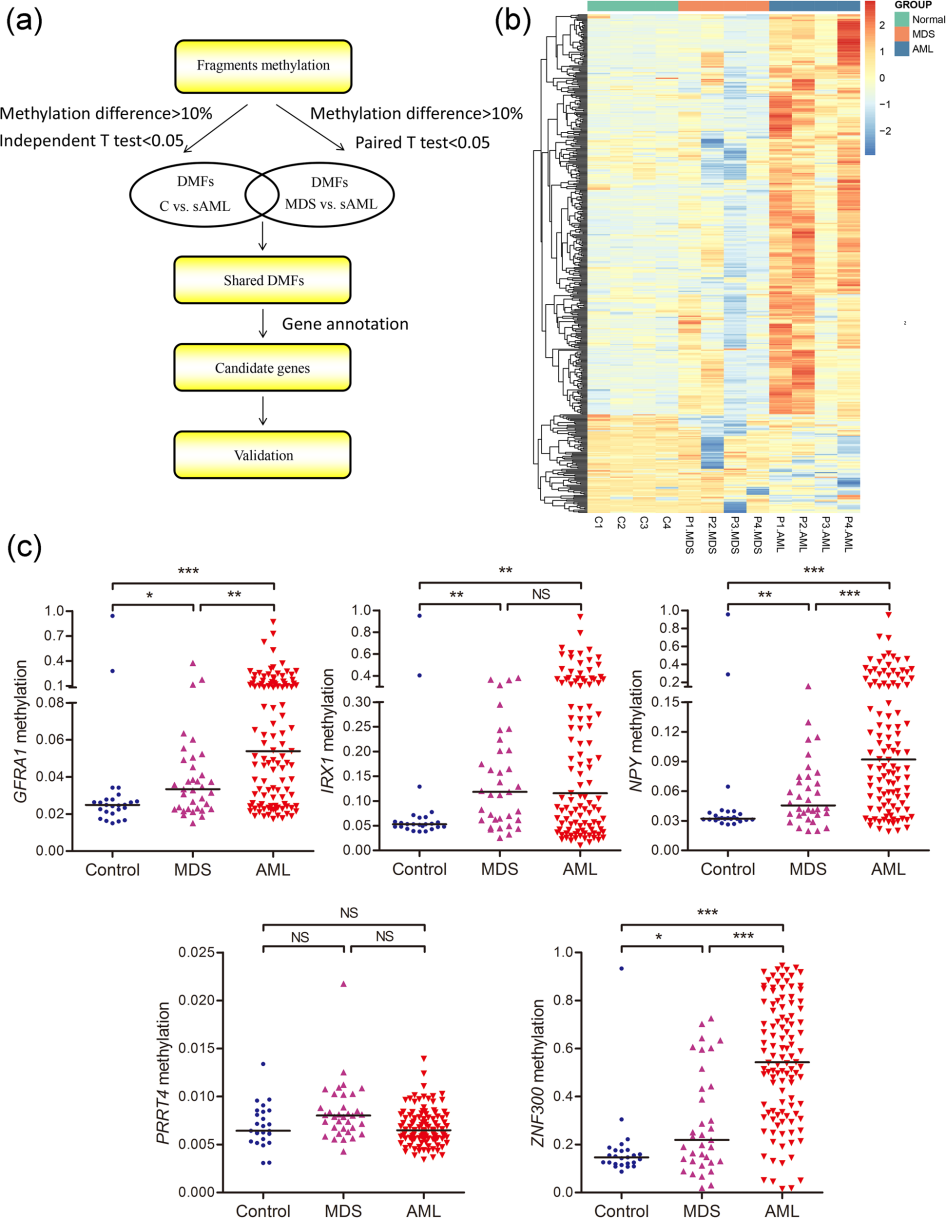
Identification and validation of differentially methylated genes during MDS progression. **a** The flowchart of the differentially methylated genes screening. The fragments that passed statistical significance (paired/independent *T* test-*P* < 0.05, and also had >10% mean methylation difference) were considered as differentially methylated fragments/genes. **b** Heatmaps summarizing differentially methylated fragments/genes in MDS progression. **c** The methylation level of the candidate genes in additional samples of controls (*n* = 25), de novo MDS (*n* = 35) and AML patients (*n* = 111) analyzed by targeted bisulfite sequencing. *P*-values were calculated using the Mann–Whitney *U*-test. NS: no significance; *P < 0.05; **P < 0.01; ***P < 0.001.

In order to identify and validate the candidate DMFs involved in MDS progression, we screened a series of DMFs (paired/independent *T* test-*P* < 0.05, and also had >10% mean methylation difference) shared in sAML vs. MDS and sAML vs. controls, and further annotated as DMGs (Fig. 5a and Table S6). A total of 486 genes were identified and five genes (*GFRA1*, *IRX1*, *NPY*, *PRRT4*, and *ZNF300*) were selected, of which may have biological functions in cancers analyzed by Coremine analysis (http://www.coremine.com/medical/#search), for further targeted bisulfite sequencing in additional 35 MDS, 111 AML patients and 25 controls using MethylTarget (GENESKY, Shanghai, China)^17^. The mean bait coverage attached 1694×, and Q30 was 75.56%^17^. As showed in Fig. 5b, the methylation level of *GFRA1*, *IRX1*, *NPY*, and *ZNF300* were markedly increased in MDS and AML patients compared with controls (Fig. 5c). Moreover, *GFRA1*, *NPY*, and *ZNF300* methylation level in AML patients also significantly higher than that in MDS patients (Fig. 5c).

### Transcriptional regulatory effects on mRNA expression of the candidate genes methylation

**Fig. 6:**
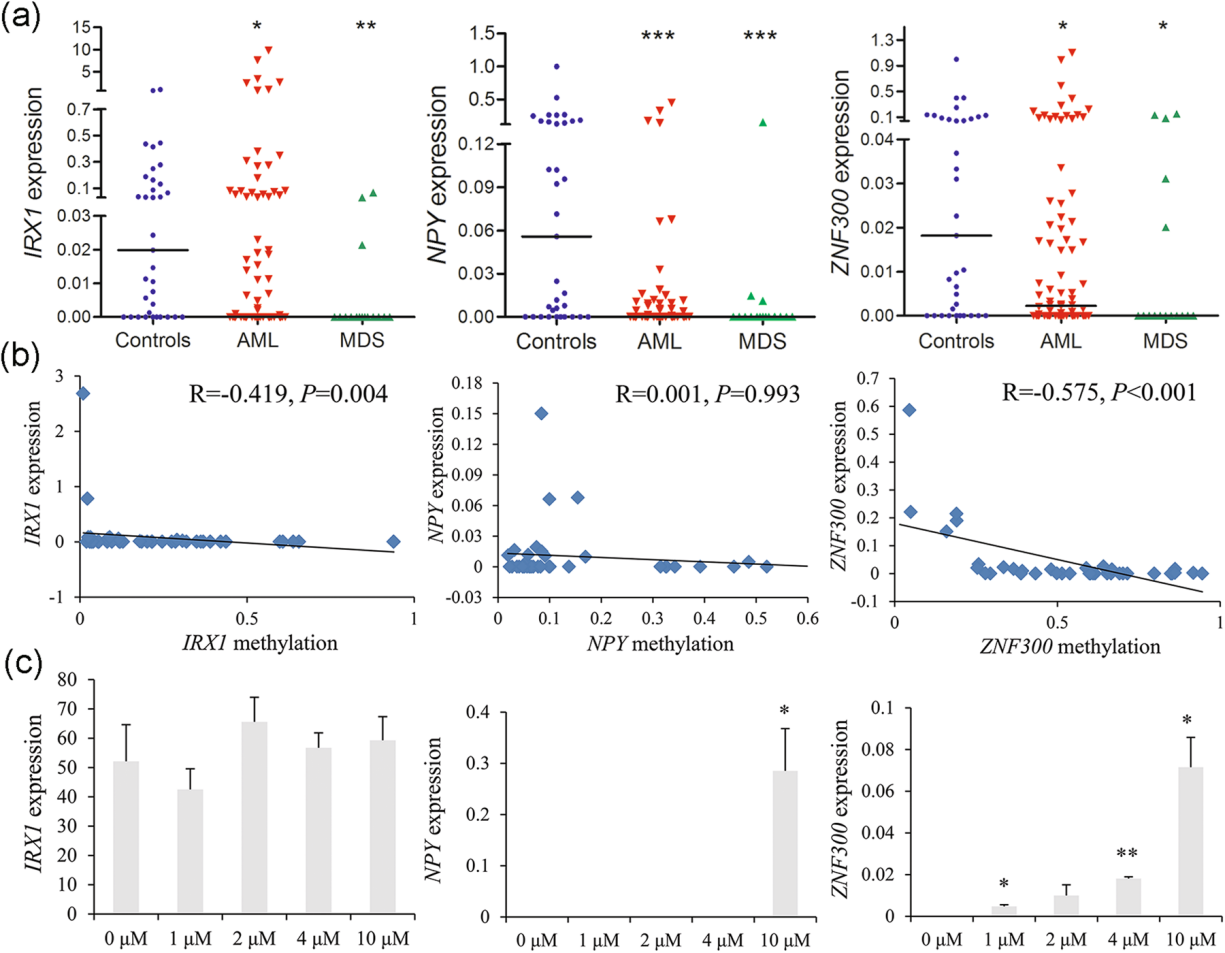
Transcriptional regulatory effects on mRNA expression of the candidate genes methylation. **a** The expression level of the candidate genes in de novo MDS and AML patients by real-time quantitative PCR. *P*-values were calculated using the Mann–Whitney *U*-test. **b** The correlation between the candidate genes methylation and genes expression. The correlation was analyzed by Spearman correlation test. **c** The expression of the candidate genes in MDS-derived AML cell line SKM-1 before and after 5-aza-dC treatment. *P*-values were calculated using the independent T-test. *P < 0.05; **P < 0.01; ***P < 0.001.

To confirm the transcriptional regulatory effects on gene expression influenced by DNA methylation, we further evaluated the candidate gene expression in clinical samples and MDS-derived AML cell-line SKM-1 before and after the treatment with demethylation agent 5-aza-dC. Overall, *IRX1*, *NPY*, and *ZNF300* expression were significantly downregulated in MDS and AML patients (Fig. 6a). Moreover, *IRX1* and *ZNF300* expression were negatively associated with gene methylation (Fig. 6b). In addition, a significantly increased expression of *ZNF300* and *NPY* were observed after 5-aza-dC demethylation treatment (Fig. 6c).

### Further confirmation of *ZNF300* methylation in a larger cohort of MDS/AML patients

**Fig. 7:**
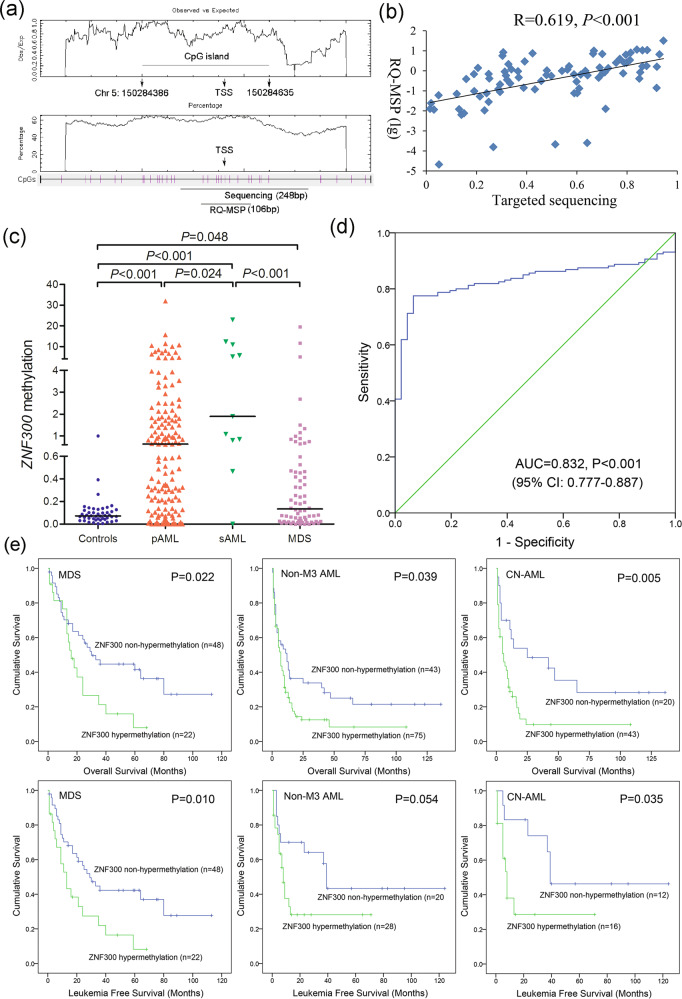
Further confirmation of *ZNF300* methylation in MDS and AML patients together with its prognostic value. **a** The genomic coordinates of *ZNF300* promoter region CpG island and primer locations. The panel plots the GC content as a percentage of the total. Each vertical bar in the bottom panel represents the presence of a CpG dinucleotide. Black horizontal bars indicate regions amplified by targeted bisulfite sequencing primer pairs and RQ-MSP primer pairs. This figure was created using CpGplot (http://emboss.bioinformatics.nl/cgi-bin/emboss/cpgplot) and Methyl Primer Express v1.0 software. TSS: transcription start site; RQ-MSP: real-time quantitative methylation-specific PCR. **b** The correlation of the candidate gene methylation results between the targeted bisulfite sequencing and RQ-MSP. The correlation was analyzed by Spearman correlation test. **c** The methylation level of the *ZNF300* in larger samples of controls (*n* = 46), de novo MDS (*n* = 70) and AML patients (*n* = 170) analyzed by targeted bisulfite sequencing. *P*-values were calculated using the Mann–Whitney *U*-test. **d** ROC curve analysis using *ZNF300* methylation for discriminating AML patients from controls. (**e**): The impact of *ZNF300* methylation on leukemia-free survival and overall survival of MDS and AML patients. Survival was analyzed through Kaplan–Meier analysis using Log-rank test.

To further investigate the value of *ZNF300* methylation in clinical diagnosis and risk/treatment assessment, we expanded the clinical samples (70 MDS and 160 AML) to explore the clinical significance of *ZNF300* methylation through a more rapid and inexpensive methodology—RQ-MSP. Primers for RQ-MSP were designed containing in the targeted sequencing primers (Fig. 7a). RQ-MSP results were highly correlated with that in the targeted sequencing (Fig. 7b). Patients with MDS and AML exhibited a markedly higher *ZNF300* methylation level than controls, and *ZNF300* methylation level in AML patients was even much higher than that in MDS patients (Fig. 7c). ROC curve analysis revealed that *ZNF300* methylation could be used to segregate AML from controls with an AUC value of 0.832 (95% CI: 0.777–0.887, Fig. 7d). For the purpose of investigating the clinical relevance of *ZNF300* methylation in MDS and AML patients, the patients were divided into two groups (*ZNF300* hypermethylated and non-hypermethylated) based on the cutoff value of 0.408 (set as “mean + 2 SD” in controls). As presented in Tables 1 and 2, *ZNF300* methylation tended to correlate with *U2AF1* and *SRSF2* mutations in MDS (*P* = 0.066 and 0.090, respectively), whereas its hypermethylation was associated with lower platelets and higher proportion of male patients/*CEBPA* mutations in AML (*P* = 0.002, 0.032, and 0.066, respectively).

**Table 1:** Comparison of clinical and laboratory features between *ZNF300* hypermethylated and non-hypermethylated MDS patients.

Patient’s features	Non-hypermethylated (*n* = 48)	Hypermethylated (*n* = 22)	*P* value
Sex (male/female)	26/22	13/9	0.798
Median age, years (range)	57.5 (27-84)	69 (28–86)	0.271
Median WBC, ×10^9^/L (range)	3.0 (1.1–44.4)	2.5 (0.6–82.4)	0.240
Median hemoglobin, g/L (range)	65 (35–140)	62 (43–107)	0.889
Median platelets, ×10^9^/L (range)	69 (0–1176)	50 (10–323)	0.475
Median BM blasts, % (range)	5 (0–19)	6 (0–18)	0.229
WHO classifications			0.840
RCUD/RARS	5	2	
RCMD/RCMD-RS	18	8	
RAEB-1	8	3	
RAEB-2	14	9	
MDS with isolated del(5q)	3	0	
IPSS scores			0.806
Low	7	2	
Int-1	23	9	
Int-2	7	5	
High	7	3	
No data	4	3	
Gene mutations			
* CEBPA* (+ /−)	2/41	0/19	1.000
* IDH1/2* (+/−)	2/41	0/19	1.000
* DNMT3A* (+/−)	0/43	1/18	0.306
* U2AF1* (+/−)	2/41	4/15	0.066
* SRSF2* (+/−)	0/43	2/17	0.090
* SF3B1* (+/−)	4/39	0/19	0.303
* SETBP1* (+/−)	1/42	0/19	1.000

*MDS* myelodysplastic syndromes, *WBC* white blood cells, *BM* bone marrow, *WHO* World Health Organization, *IPSS* International Prognostic Scoring System.

**Table 2:** Comparison of clinical and laboratory features between *ZNF300* hypermethylated and non-hypermethylated AML patients.

Patient’s features	Non-hypermethylated (*n* = 63)	Hypermethylated (*n* = 97)	*P* value
Sex, male/female	31/32	65/32	0.032
Median age, years (range)	57 (18–85)	55 (18–86)	0.522
Median WBC, ×10^9^/L (range)	11.75 (0.9–528.0)	18.7 (0.3–201.0)	0.261
Median hemoglobin, g/L (range)	83 (42–135)	76 (32–144)	0.273
Median platelets, ×10^9^/L (range)	53 (3–447)	32 (5–415)	0.002
Median BM blasts, % (range)	56.64 (5.5*–97.5)	49.25 (1.0*–99.0)	0.881
FAB classifications			0.670
M0	0	2	
M1	2	8	
M2	23	39	
M3	14	15	
M4	13	17	
M5	8	11	
M6	3	3	
No data	0	2	
Karyotypes			0.366
normal	25	46	
t(8;21)	2	9	
inv(16)	0	1	
t(15;17)	14	13	
+8	3	2	
−7/7q−	0	1	
t(9;22)	1	1	
11q23	0	2	
complex	10	7	
others	4	9	
No data	4	6	
Gene mutations			
* CEBPA* (+/−)	2/53	10/61	0.066
* NPM1* (+/−)	5/50	9/62	0.580
* FLT3*-ITD (+/−)	4/51	7/64	0.755
* C-KIT* (+/−)	3/52	4/67	1.000
* N/K-RAS* (+/−)	4/51	8/63	0.549
* IDH1/2* (+/−)	3/52	2/69	0.652
* DNMT3A* (+/−)	4/51	4/67	0.728
* U2AF1* (+/−)	0/55	3/68	0.256
* SRSF2* (+/−)	2/53	2/69	1.000
* SETBP1* (+/−)	0/55	2/69	0.504
CR, total AML (+/−)	31/25	30/50	0.054
CR, non-M3 AML (+/−)	20/23	20/48	0.073
CR, CN-AML (+/−)	12/8	12/27	0.049

*WBC* white blood cells, *BM* bone marrow, *FAB* French-American-British classification, CR complete remission.

*Patients’ blasts less than 20% with t(15;17) cytogenetic aberrations.

### Prognostic effect of *ZNF300* methylation in MDS/AML patients

In MDS patients, Kaplan–Meier analyses indicated that *ZNF300* hypermethylated cases exhibited significantly shorter OS and LFS time than *ZNF300* non-hypermethylated cases (*P* = 0.022 and 0.010, respectively) (Fig. 7e). Moreover, Cox regression analyses also confirmed the independent prognostic effect of *ZNF300* methylation on OS and LFS (*P* = 0.037 and 0.038, respectively) (Table S7). In AML patients, we first evaluated the association between *ZNF300* methylation and CR. Cases with *ZNF300* hypermethylation had lower CR rate as compared with cases with *ZNF300* non-hypermethylation among total and non-M3 AML as well as cytogenetically normal AML (CN-AML) subgroups (*P* = 0.054, 0.073, and 0.049, respectively) (Table 2). Due to the significant associations of *ZNF300* methylation with CR among CN-AML, Logistic regression analyses were further performed to confirm the effect of *ZNF300* methylation on CR. After adjusting for the known factors, *ZNF300* hypermethylation was a negative independent risk factor affecting CR in CN-AML patients (*P* = 0.015) (Table S8). Next, Kaplan–Meier analyses showed that *ZNF300* hypermethylated cases exhibited shorter OS and LFS time compared with *ZNF300* non-hypermethylated cases in both non-M3 AML (*P* = 0.039 and 0.054, respectively) and CN-AML (*P* = 0.005 and 0.035, respectively) (Fig. 7e). Additionally, Cox regression analysis revealed that *ZNF300* hypermethylation was an independent prognostic factor affecting OS in both non-M3 AML and CN-AML patients (*P* = 0.013 and 0.018, respectively) (Table S9).

### Biological role of *ZNF300* in MDS-derived AML cell-line SKM-1

**Fig. 8:**
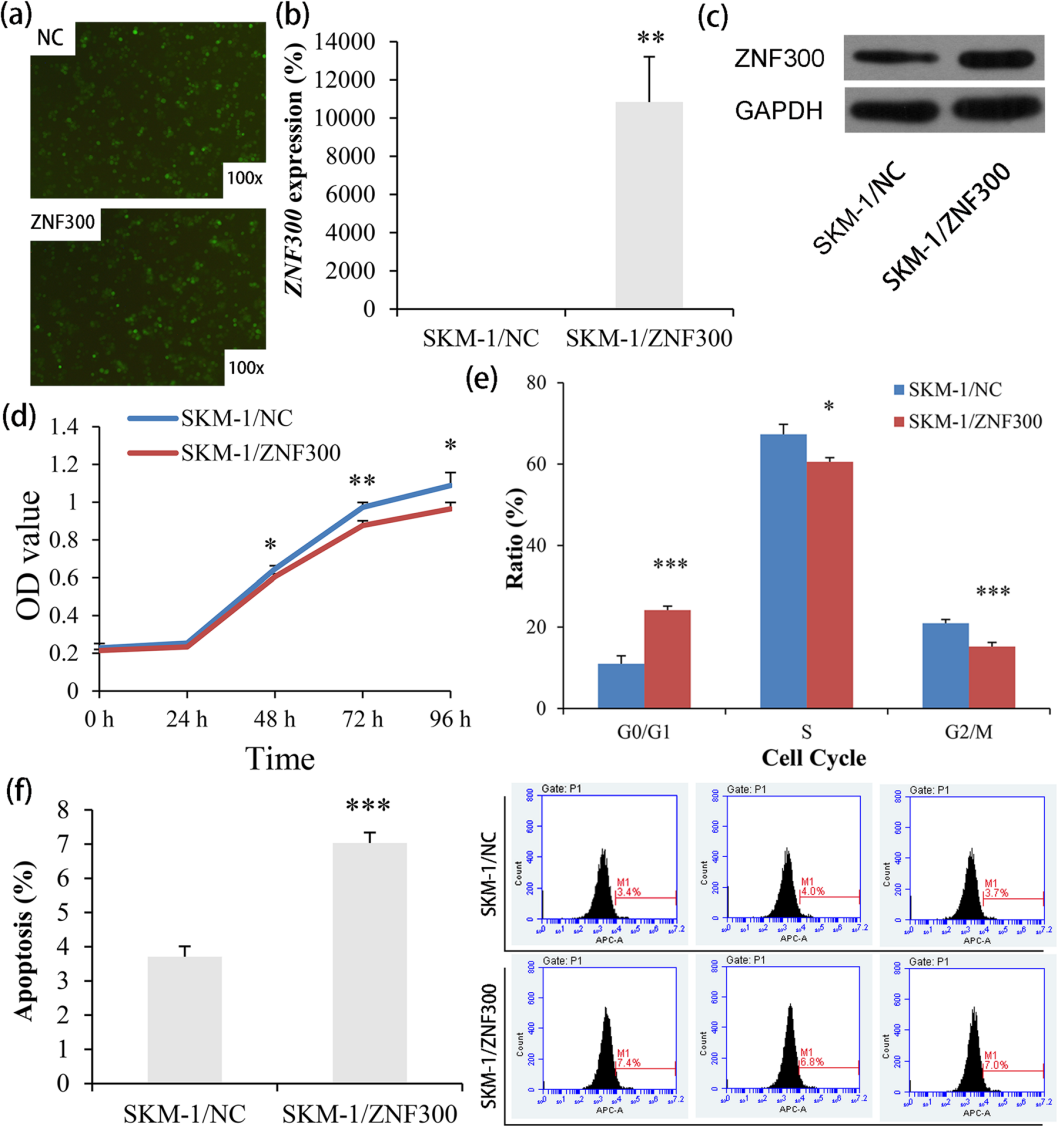
Biological functions of *ZNF300* in MDS-derived AML cell line SKM-1. **a** Fluorescence detection after LV-NC and LV-*ZNF300* transfection in SKM-1. **b** Confirmation of *ZNF300* mRNA level in SKM-1 after transfection by real-time quantitative PCR. **c** Confirmation of *ZNF300* overexpression in SKM-1 after transfection by western blot. **d** The proliferation ability in SKM-1 affected by *ZNF300* overexpression. **e** The cell cycle in SKM-1 affected by *ZNF300* overexpression. **f** The apoptosis ability in SKM-1 affected by *ZNF300* overexpression. P-values were calculated using the independent T-test. *P < 0.05; **P < 0.01; ***P < 0.001.

To investigate the underlying role of *ZNF300* during MDS progression, we carried out gain-of-function experiments in MDS-derived AML cell-line SKM-1 in vitro. Firstly, we successfully established SKM-1 cells stably overexpressing *ZNF300* by Lv-*ZNF300* infection, which was determined by fluorescence, RQ-PCR, and western blot (Fig. 8a–c). Notably, SKM-1 cells overexpressing *ZNF300* exhibited significantly lower growth rate than those without *ZNF300* overexpression (Fig. 8d), and could cause G0/G1 arrest as well (Fig. 8e). Conversely, the apoptosis of SKM-1 cells were significantly promoted after *ZNF300* overexpression (Fig. 8f).

## Discussion

Recent studies have made great progresses in understanding of the mechanism underlying MDS progression. Cytogenetic abnormalities, such as -7/7q-, +8, 6q-, 11q-, i(7q), 11q-, t(7;9), i(9q), and complex karyotypes play crucial roles in MDS evolution^22,23^. With the rapid advances in sequencing technologies, somatic mutations were also identified to contribute to disease progression in MDS, such as genes encoding transcription factors (*ETV6* and *TP53*), epigenetic regulators [*DNMT3A* (methylation), *TET2* and *IDH1/2* (hydroxymethylation), *EZH2* and *ASXL1* (histones modifications)], and splicing factors (*U2AF1*)^22,23^. Recently, Xu et al. by whole-exome and targeted sequencing revealed that *ROBO1/2* mutations acted as progression-related drivers in MDS^7^. In addition to genetic alterations, epigenetic modifications, especially for DNA methylation were also reported to be participated in cancer progression including MDS^24–26^. Previous studies almost focused on the single gene change during MDS progression, such as *CDKN2B*, *SOCS1*, *NR4A2*, *ABAT*, *ID4, GPX3*, *SOX30*, and so on^9–11,27–30^. However, few studies demonstrated the whole-genome DNA methylation alterations during MDS progression.

In this study, we for the first time used RRBS in four paired MDS/sAML patients to identify the methylation-associated epigenetic drivers underlying MDS progression. We identified and verified that whole-genome DNA hypermethylation was a frequent phenomenon during MDS progression. Moreover, as analyzed in details, the number of hypermethylated fragments was larger than hypomethylated fragments during MDS progression, but not presented during MDS incidence. Previously studies also exhibited that aberrant methylation existed in every MDS sample, on average affecting 91 of 1505 CpG loci in early MDS and 179 of 1505 loci after blast transformation^26^. Figueroa et al. reported that abnormal methylation in MDS and sAML tended to affect particular chromosomal regions, occurred more frequently in Alupoor genes, and included prominent involvement of genes involved in the WNT and MAPK signaling pathways^25^. In the current study, although some of these genes methylation (such as *S0X17* and *RAP1GAP*) were previously reported in MDS^31,32^, the majority of them were unknown so far. Notably, several genes were reported to be methylated in other hematologic malignancies or solid tumors, such as *HOXD11*, *GBX2*, *CRMP1*, *RBM47*, *NHLRC1*, *WNT2*, *TUSC3*, *NRG1*, *TSPYL5*, *CNTFR*, *NR4A3*, *PHOX2A*, *KCNA5*, *PTGDR*, *HS3ST2*, *CLDN7*, *CPT1C*, and *NKX2-4*^33–48^. Based on these findings, we further selected the unreported hypermethylated genes with potential roles involving in MDS progression for further validation. The targeted bisulfite sequencing in additional expanded de novo MDS and AML samples identified *GFRA1*, *IRX1*, *NPY*, and *ZNF300* were frequently hypermethylated in these patients. Moreover, *GFRA1*, *NPY*, and *ZNF300* methylation were more frequently happened in AML patients than in MDS patients, suggesting that the aberrant hypermethylation of these genes played vital roles in MDS transformation.

Assignment of the DMFs to genomic regions demonstrated that DMFs were mostly located in or around CpG islands, suggesting that these DMFs might play a role in the regulation of transcriptional gene expression as methylation of CpG islands located in promoter were strongly associated with gene silencing. Nevertheless, it was a pity that we did not obtain enough RNA samples corresponding to these paired MDS/sAML patients for genome-wide gene expression sequencing in our study. We only detected the expression level of the selected genes (*GFRA1*, *IRX1*, *NPY*, and *ZNF300*) in de novo MDS and AML samples by conducting RQ-PCR. *IRX1*, *NPY* and *ZNF300* expression were significantly reduced in de novo MDS and AML patients, and only *IRX1* and *ZNF300* expression were associated with their gene methylation analyzed by targeted bisulfite sequencing. Additionally, epigenetic studies further confirmed that *ZNF300* expression was significantly associated with *ZNF300* methylation in MDS. These results, in general, suggested that *ZNF300* methylation functioned in MDS progression mainly via downregulating *ZNF300* expression.

The direct role and clinical implication of *ZNF300* in MDS and AML remain poorly investigated. Cai et al. demonstrated that *ZNF300* knockdown inhibited forced megakaryocytic differentiation by phorbol and erythrocytic differentiation by arabinofuranosyl cytidine in chronic myeloid leukemia cell line K562^49^. Our study further exhibited the direct role of *ZNF300* in MDS-derived AML cell-line SKM-1 with potential anti-proliferative and pro-apoptotic effects. In addition to hematologic malignancies, *ZNF300* gene overexpression enhanced growth and metastasis of cancer cells through activating NF-κB pathway in cervical cancer^50^. Moreover, we for the first time determined clinical implication of *ZNF300* methylation in de novo MDS and AML patients, and found that *ZNF300* methylation was a potential biomarker helpful for diagnosis in AML. Furthermore, *ZNF300* methylation could act as an independent prognostic biomarker affecting LFS, OS, and CR in MDS and/or AML. Taken all the results together, it is inferred that *ZNF300* may act as a potential therapeutic target in MDS and AML against disease progression, and using *ZNF300*-based targeted therapy could improve the clinical outcome for MDS and AML patients. Since this was the first report regarding *ZNF300* in MDS and AML, prospective studies and integrative analysis are needed before the promising biomarkers can be routinely used for risk stratification and planning therapy in MDS and AML.

In summary, by using next generation sequencing, it was apparently revealed that genome-wide DNA hypermethylation were frequent events during MDS progression. Among these changes, *ZNF300* methylation through regulating *ZNF300* expression acted as an epigenetic driver in MDS progression. These findings provided a theoretical basis for the usage of demethylation drugs in MDS patients against disease progression and opened up new insights for targeted therapy in MDS.

## Supplementary information

Supplementary Figure Legends

Figure S1

Table S3

Table S4

Table S5

Table S6
