# Supplementary material for: Genome-wide methylation sequencing identifies progression-related epigenetic drivers in myelodysplastic syndromes
Source: Cell Death Dis. 2020 Nov 20;11(11):997. doi: 10.1038/s41419-020-03213-2 (PMC7679421; doi:10.1038/s41419-020-03213-2)
Supplement: Supplementary file 1 — Supplementary Figure Legends [file 41419_2020_3213_MOESM1_ESM.docx]

**Figure S1. Whole-genome methylation patterns and clustering of controls and paired MDS/sAML patients.** Hierarchical clustering of 4 controls and 4 paired MDS/sAML patients based on CpG site methylation separated MDS/sAML patients from controls.

**Table S1. Clinic-pathologic characteristics of four paired MDS/sAML patients used for RRBS**

| Patients | Age/Sex | MDS stage | | | | Progression time (months) | sAML stage | | | |
| --- | --- | --- | --- | --- | --- | --- | --- | --- | --- | --- |
|  |  | WBC/HB/PLT | Blasts | Karyotype | Treatment |  | FAB | WBC/HB/PLT | Blasts | Karyotype |
| P1 | 41/Male | 2.49/91/27 | 16.5% | Normal | IAG | 25 | M6 | 6.07/170/172 | 20% | No data |
| P2 | 36/Male | 1.35/116/12 | 12% | Normal | Supportive | 20 | M6 | 0.36/60/10 | 25% | 47,XX,+21 |
| P3 | 69/Female | 3.43/65/38 | 11% | Complex | Supportive | 11 | M6 | No data | 20.5% | Normal |
| P4 | 74/Female | 5.02/61/72 | 18% | 47,XX,+8 | CAG/C | 20 | M4a | 0.9/63/3 | 55.3% | No data |

WBC: White blood cells (×10^9^/L); HB: Hemoglobin (g/L); PLT: Platelets (×10^9^/L); C: Cytarabine; IAG: Cytarabine+Idarubicin+G-CSF; CAG: Cytarabine+Aclarithromycin+G-CSF.

**Table S2. Primers used for MethylTarget sequencing, RQ-PCR, and RQ-MSP.**

| Primers | Primer sequence (5’to 3’) |
| --- | --- |
| MethylTarget sequencing |  |
| *GFRA1-*F | TTTTTATATTTTYGTTYGGTGTTTAGG |
| *GFRA1*-R | CCCTCTTCCCCACTCCTTCT |
| *IRX1*-F | GGAGTTGTTAAAGAGGGAGAAAA |
| *IRX1*-R | CAAACTCRCCTAACTCRACCAAAAC |
| *NPY*-F | GGGGAGTAGAGGAATAAGTGGA |
| *NPY*-R | AACATAATACTTACCRCCCAACCAC |
| *PRRT4*-F | TTTATATTYGGGYGGGTAGGTAGG |
| *PRRT4*-R | AAATACCATCCTAAAACAAACTACCA |
| *ZNF300*-F | GGTTTTGTTTAGGAAGTAATATGGTTGTT |
| *ZNF300*-R | ATTCACTCCAACTTCCTCTTCAATC |
|  |  |
| RQ-PCR |  |
| *GFRA1*-F | ACAGGTCGGCGTACATCAC |
| *GFRA1*-R | GGAGGCAGTCAGCGTAGTTTT |
| *IRX1*-F | GGGGCACTCAATGGAGACAAG |
| *IRX1*-R | TACCGGCTGGAAGGGCGACT |
| *NPY*-F | CCTGTCCCTGCTCGTGT |
| *NPY*-R | TCTGGGCTGGATCGTTT |
| *PRRT4*-F | CCAGGTGAAACTACATCCACAAGC |
| *PRRT4*-R | CCCTCGCGTTCAGGCAAT |
| *ZNF300*-F | GCAGATGGGAGACAAGAC |
| *ZNF300*-R | AACAACTCGGTAGGTCAAT |
|  |  |
| RQ-MSP primers |  |
| *ZNF300*-MF | GTTGTTTTCGTATGGGGTC |
| *ZNF300*-MR | CGCTACCGCTATAATACAACC |
| *ZNF300*-UF | GAGTTGTTTTTGTATGGGGTT |
| *ZNF300*-UR | CACTACCACTATAATACAACCCA |

RQ-PCR: real-time quantitative PCR; RQ-MSP: real-time quantitative methylation-specific PCR.

**Table S3. Reduced Representation Bisulfite Sequencing (RRBS) data quality of the 4 controls and 4 paired MDS/sAML patients.**

Presented as Excel file.

**Table S4. Differentially methylated genes (DMGs) involved in MDS pathogenesis.**

Presented as Excel file.

**Table S5. Differentially methylated genes (DMGs) may be involved in MDS progression.**

Presented as Excel file.

**Table S6. Further identification of differentially methylated genes (DMGs) involved in MDS progression.**

Presented as Excel file.

**Table S7. Cox regression analyses of** **variables for overall and leukemia-free survival in MDS patients**

| Variables | | Univariate analyses | | | | | Multivariate analyses | | | |
| --- | --- | --- | --- | --- | --- | --- | --- | --- | --- | --- |
|  |  | Hazard ratio (95% CI) | | | *P* value | | Hazard ratio (95% CI) | | *P* value | |
| Overall Survival | | | | | | | | | | |
| *ZNF300* methylation | | 1.996 (1.088-3.661) | | | 0.025 | | 2.053 (1.043-4.042) | | 0.037 | |
| Age | | 2.019 (1.112-3.667) | | | 0.021 | | 2.259 (1.142-4.469) | | 0.019 | |
| IPSS risks | | 1.412 (1.006-1.982) | | | 0.046 | | 1.419 (0.925-2.177) | | 0.109 | |
| *CEBPA* mutation | | 0.045 (0.000-15.472) | | | 0.298 | |  | |  | |
| *IDH1/2* mutation | | 0.499 (0.068-3.642) | | | 0.493 | |  | |  | |
| *DNMT3A* mutation | | 1.653 (0.224-12.180) | | | 0.622 | |  | |  | |
| *U2AF1* mutation | | 0.835 (0.297-2.351) | | | 0.733 | |  | |  | |
| *SF3B1* mutation | | 1.185 (0.284-4.938) | | | 0.816 | |  | |  | |
| *SRSF2* mutations | | 2.474 (0.582-10.520) | | | 0.220 | |  | |  | |
| *SETBP1* mutations | | 11.610 (1.356-99.410) | | | 0.025 | | 28.573 (2.948-276.945) | | 0.004 | |
| Leukemia-free Survival | | | | | | | | | | |
| *ZNF300* methylation | | 2.137 (1.171-3.901) | 0.013 | | 2.070 (1.040-4.119) | | 0.038 | |  |  |
| Age | | 2.122 (1.166-3.862) | 0.014 | | 2.468 (1.229-4.958) | | 0.011 | |  |  |
| IPSS risks | | 1.476 (1.041-2.092) | 0.029 | | 1.460 (0.947-2.251) | | 0.087 | |  |  |
| *CEBPA* mutation | | 0.045 (0.000-15.544) | 0.298 | |  | |  | |  |  |
| *IDH1/2* mutation | | 0.501 (0.069-3.652) | 0.495 | |  | |  | |  |  |
| *DNMT3A* mutation | | 2.193 (0.295-16.284) | 0.443 | |  | |  | |  |  |
| *U2AF1* mutation | | 0.898 (0.319-2.529) | 0.839 | |  | |  | |  |  |
| *SF3B1* mutation | | 1.105 (0.265-4.596) | 0.891 | |  | |  | |  |  |
| *SRSF2* mutations | | 2.572 (0.608-10.888) | 0.199 | |  | |  | |  |  |
| *SETBP1* mutations | | 9.599 (1.155-79.756) | 0.036 | | 29.714 (3.110-283.925) | | 0.003 | |  |  |

IPSS: International Prognostic Scoring System. Variables including age (≤60 vs. >60 years old), IPSS scores (Low vs. Int-1 vs. Int-2 vs. High), *ZNF300* methylation (non-hypermethylated vs. hypermethylated), and gene mutations (mutant vs. wild-type). Multivariate analysis includes variables with *P*<0.100 in univariate analysis.

**Table S8. Logistic regression analyses of variables for complete remission in CN-AML patients**

| Variables | Univariate analysis | | Multivariate analysis | |
| --- | --- | --- | --- | --- |
|  | odds ratio (95% CI) | *P* value | odds ratio (95% CI) | *P* value |
| *ZNF300* methylation | 0.296 (0.096-0.911) | 0.034 | 0.198 (0.053-0.734) | 0.015 |
| Age | 0.243 (0.080-0.743) | 0.013 | 0.172 (0.048-0.615) | 0.007 |
| WBC | 0.421 (0.143-1.238) | 0.116 |  |  |
| *CEBPA* mutations | undetermined | 0.999 |  |  |
| *NPM1* mutations | 1.618 (0.376-6.960) | 0.518 |  |  |
| *FLT3*-ITD mutations | 0.767 (0.116-5.059) | 0.783 |  |  |
| *C-KIT* mutations | undetermined | 1.000 |  |  |
| *N/K-RAS* mutations | 0.420 (0.073-2.418) | 0.331 |  |  |
| *IDH1/2* mutations | 1.200 (0.155-9.301) | 0.861 |  |  |
| *DNMT3A* mutations | 0.767 (0.116-5.059) | 0.783 |  |  |
| *U2AF1* mutations | undetermined | 0.999 |  |  |
| *SRSF2* mutations | undetermined | 0.999 |  |  |
| *SETBP1* mutations | undetermined | 1.000 |  |  |

Variables including *ZNF300* methylation (hypermethylation vs. non-hypermethylation), age (≤60 vs. >60 years), WBC (≥30×10^9^ vs. <30×10^9^ /L), and gene mutations (mutant vs. wild-type). Multivariate analysis includes variables with *P*<0.200 in univariate analysis.

**Table S9. Cox regression analyses of variables for overall survival in non-M3 AML and CN-AML patients**

| Variables | Univariate analysis | | Multivariate analysis | | |  |
| --- | --- | --- | --- | --- | --- | --- |
|  | hazard ratio (95% CI) | *P* value | hazard ratio (95% CI) | *P* value | |  |
| Non-M3 AML | | | | | |  |
| *ZNF300* methylation | 1.545 (1.001-2.386) | 0.049 | 1.790 (1.128-2.841) | 0.013 | |  |
| Age | 2.247 (1.492-3.383) | 0.000 | 2.062 (1.351-3.147) | 0.001 | |  |
| WBC | 1.880 (1.249-2.831) | 0.002 | 1.580 (1.046-2.388) | 0.030 | |  |
| Cytogenetic risks | 1.536 (1.176-2.006) | 0.002 | 1.571 (1.174-2.102) | 0.002 | |  |
| *CEBPA* mutations | 1.689 (0.806-3.541) | 0.165 |  |  | |  |
| *NPM1* mutations | 0.806 (0.386-1.684) | 0.566 |  |  | |  |
| *FLT3*-ITD mutations | 1.267 (0.581-2.764) | 0.551 |  |  | |  |
| *C-KIT* mutations | 0.796 (0.250-2.533) | 0.700 |  |  | |  |
| *N/K-RAS* mutations | 0.975 (0.483-1.969) | 0.944 |  |  | |  |
| *IDH1/2* mutations | 0.730 (0.227-2.342) | 0.597 |  |  | |  |
| *DNMT3A* mutations | 1.475 (0.676-3.221) | 0.329 |  |  | |  |
| *U2AF1* mutations | 2.488 (0.764-8.098) | 0.130 |  |  | |  |
| *SRSF2* mutations | 2.031 (0.732-5.633) | 0.173 |  |  | |  |
| *SETBP1* mutations | 0.591 (0.082-4.266) | 0.602 |  |  | |  |
| CN-AML | | | | | |  |
| *ZNF300* methylation | 2.461 (1.262-4.799) | 0.008 | 2.436 (1.164-5.096) | | 0.018 | |
| Age | 2.570 (1.428-4.625) | 0.002 | 2.813 (1.385-5.711) | | 0.004 | |
| WBC | 2.002 (1.124-3.565) | 0.018 | 1.969 (1.014-3.822) | | 0.045 | |
| *CEBPA* mutations | 6.590 (2.017-21.531) | 0.002 | 4.876 (1.423-16.704) | | 0.012 | |
| *NPM1* mutations | 0.568 (0.220-1.470) | 0.244 |  | |  | |
| *FLT3*-ITD mutations | 0.947 (0.335-2.675) | 0.918 |  | |  | |
| *C-KIT* mutations | 0.046 (0.000-85.914) | 0.423 |  | |  | |
| *N/K-RAS* mutations | 1.001 (0.386-2.594) | 0.998 |  | |  | |
| *IDH1/2* mutations | 0.481 (0.114-2.036) | 0.320 |  | |  | |
| *DNMT3A* mutations | 1.349 (0.523-3.476) | 0.536 |  | |  | |
| *U2AF1* mutations | 2.143 (0.498-9.218) | 0.306 |  | |  | |
| *SRSF2* mutations | 3.228 (0.732-14.235) | 0.122 |  | |  | |
| *SETBP1* mutations | 5.153 (0.653-40.686) | 0.120 |  | |  | |

Variables including *ZNF300* methylation (hypermethylation vs. non-hypermethylation), age (≤60 vs. >60 years), WBC (≥30×10^9^ vs. <30×10^9^ /L), and gene mutations (mutant vs. wild-type). Multivariate analysis includes variables with *P*<0.100 in univariate analysis.
