# Supplementary figures and images for: Genome-wide methylation sequencing identifies progression-related epigenetic drivers in myelodysplastic syndromes
Source: Cell Death Dis. 2020 Nov 20;11(11):997. doi: 10.1038/s41419-020-03213-2 (PMC7679421; doi:10.1038/s41419-020-03213-2)

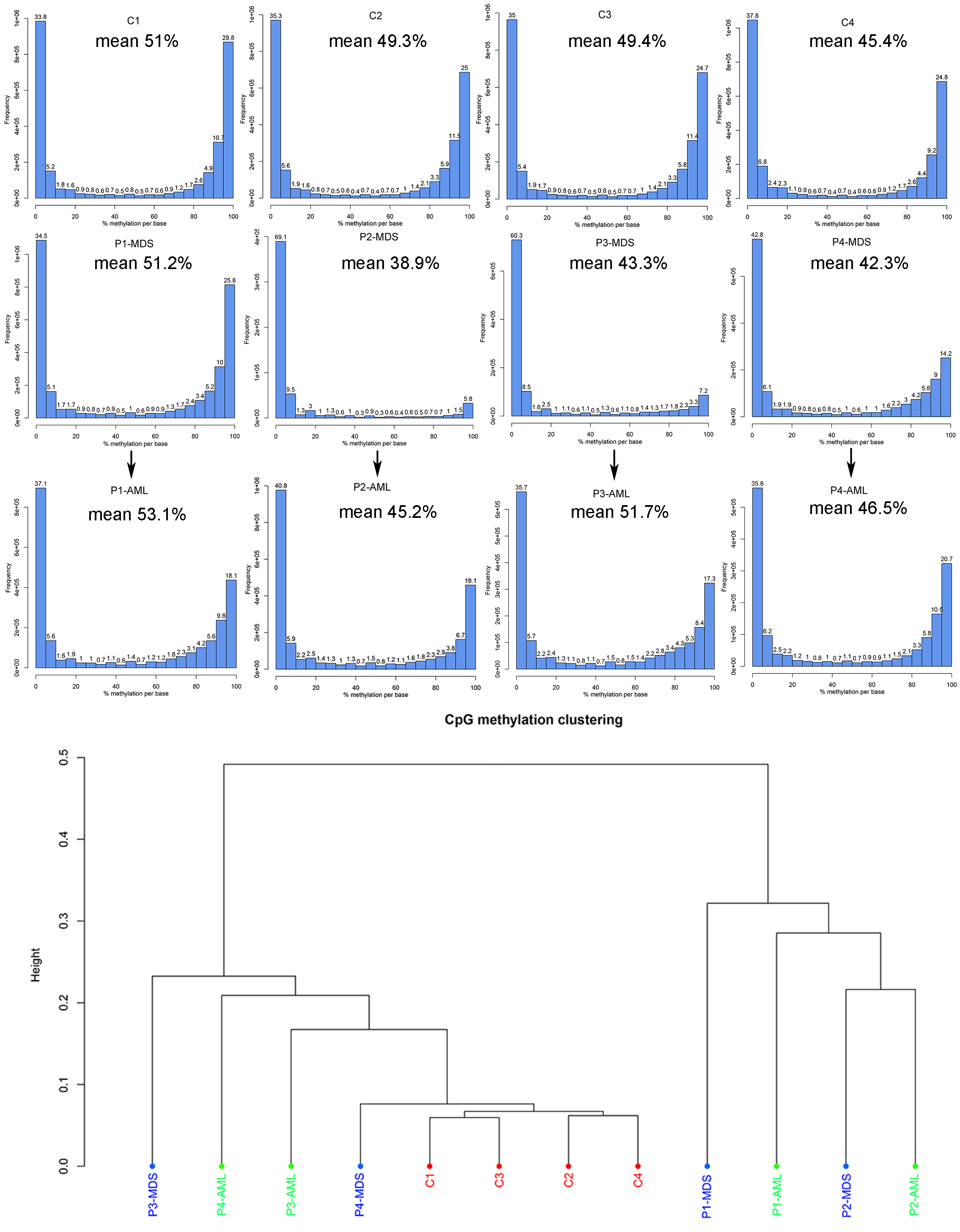

Supplement: Supplementary file 2 — Figure S1 [file 41419_2020_3213_MOESM2_ESM.tif]
